# Beyond Risk Compensation: Clusters of Antiretroviral Treatment (ART) Users in Sexual Networks Can Modify the Impact of ART on HIV Incidence

**DOI:** 10.1371/journal.pone.0163159

**Published:** 2016-09-22

**Authors:** Wim Delva, Stéphane Helleringer

**Affiliations:** 1 The South African Department of Science and Technology-National Research Foundation (DST-NRF) Centre of Excellence in Epidemiological Modelling and Analysis (SACEMA), Stellenbosch University, Stellenbosch, South Africa; 2 International Centre for Reproductive Health, Ghent University, Ghent, Belgium; 3 Center for Statistics, Hasselt University, Diepenbeek, Belgium; 4 Rega Institute for Medical Research, KU Leuven, Leuven, Belgium; 5 Institute of Integrative Biology, ETH Zurich, Zurich, Switzerland; 6 Bloomberg School of Public Health, Johns Hopkins University, Baltimore, Maryland, United States of America; University of Toronto, CANADA

## Abstract

**Introduction:**

Concerns about risk compensation—increased risk behaviours in response to a perception of reduced HIV transmission risk—after the initiation of ART have largely been dispelled in empirical studies, but other changes in sexual networking patterns may still modify the effects of ART on HIV incidence.

**Methods:**

We developed an exploratory mathematical model of HIV transmission that incorporates the possibility of ART clusters, i.e. subsets of the sexual network in which the density of ART patients is much higher than in the rest of the network. Such clusters may emerge as a result of ART homophily—a tendency for ART patients to preferentially form and maintain relationships with other ART patients. We assessed whether ART clusters may affect the impact of ART on HIV incidence, and how the influence of this effect-modifying variable depends on contextual variables such as HIV prevalence, HIV serosorting, coverage of HIV testing and ART, and adherence to ART.

**Results:**

ART homophily can modify the impact of ART on HIV incidence in both directions. In concentrated epidemics and generalized epidemics with moderate HIV prevalence (≈ 10%), ART clusters can enhance the impact of ART on HIV incidence, especially when adherence to ART is poor. In hyperendemic settings (≈ 35% HIV prevalence), ART clusters can reduce the impact of ART on HIV incidence when adherence to ART is high but few people living with HIV (PLWH) have been diagnosed. In all contexts, the effects of ART clusters on HIV epidemic dynamics are distinct from those of HIV serosorting.

**Conclusions:**

Depending on the programmatic and epidemiological context, ART clusters may enhance or reduce the impact of ART on HIV incidence, in contrast to serosorting, which always leads to a lower impact of ART on HIV incidence. ART homophily and the emergence of ART clusters should be measured empirically and incorporated into more refined models used to plan and evaluate ART programmes.

## Introduction

Early initiation of antiretroviral therapy (ART) significantly improves the survival of persons living with HIV (PLWH) and may reduce HIV transmission to uninfected partners by more than 90% [1–3]. Some mathematical models suggest that treatment-as-prevention (TasP) programmes could lead to HIV elimination, even in some of the most severely affected settings [4, 5]. International organizations including PEPFAR, the International AIDS Society, UNAIDS, The Global Fund to Fight AIDS, Tuberculosis, and Malaria, and the International Association of Providers of AIDS Care have thus placed ART scale-up at the centre of their approach to achieving an “AIDS-free generation” [6–8].

But the clinical efficacy of ART in preventing HIV transmission may not translate into an equally large population-level impact on HIV incidence in real-life settings [9]. In particular, for TasP programmes to be effective, a high proportion of PLWH must 1) be diagnosed, 2) be linked to care, 3) remain in care, and 4) adhere to ART. An ambitious programme of implementation science now seeks to improve each step of this HIV treatment cascade [10–17].

**Fig 1:**
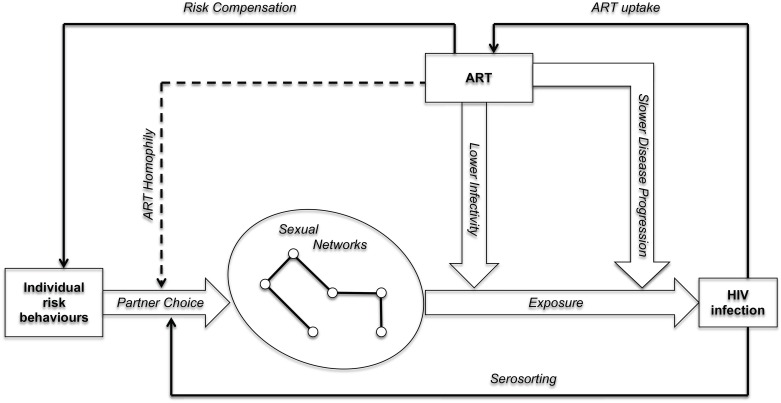
Causal pathways linking the availability of ART to HIV incidence within a population. Notes: wide arrows represent direct pathways, whereas solid narrow arrows represent feedback loops that have been considered in the literature on treatment-as-prevention. The dotted arrow represents ART homophily, another causal feedback loop that has not been considered in the literature on treatment-as-prevention.

The effects of ART on HIV incidence may also depend on changes in sexual networking dynamics during the course of ART scale-up. The Health Belief Model [18] suggests that “risk compensation” is one mechanism through which negative feedback loops between ART and HIV incidence may emerge (Fig 1). Risk compensation occurs when people increase their individual risk behaviours (e.g. having more sexual partners) in response to the increased availability of interventions to prevent and manage HIV infection [19–23]. Empirical studies of risk compensation have however generally concluded that there was limited risk compensation after ART initiation. Neither ART patients, nor HIV-negative individuals living in communities where ART becomes available, seem to increase the number of their sexual partners, for example [24–26].

### ART homophily in sexual networks

Even if ART does not lead to risk compensation, other feedback loops may emerge in the complex causal system linking ART scale-up and HIV incidence. In particular, increasing ART availability may affect the process of partnership selection/dissolution through which sexual networks are formed (Fig 1). For example, during the course of ART scale-up, ART patients may increasingly seek to form new relationships with other ART patients. Similarly, relationships between two ART patients may be less likely to dissolve than other relationships in which only one of the two partners is an ART patient. We call this phenomenon “ART homophily”.

Qualitative studies have documented several psychosocial mechanisms that may lead to such relationship dynamics. For an ART patient, ART homophily may indeed reduce fear for further HIV transmission [27] and ease anxiety about HIV status disclosure [28]. ART homophily also gives patients direct access to emotional support [29] and facilitates sharing of coping strategies during episodes of drug- induced side effects or HIV treatment fatigue [30]. In addition, ART patients may share common life histories, e.g., being widowed or having lost a previous partner. Finally, in a number of ART programmes, ART patients frequently interact with each other during dedicated ART clinics, in support groups or in various income-generating activities [31]. This increased social proximity may lead to emotional closeness, and may provide additional opportunities to form new sexual partnerships with others who are also ART patients.

ART homophily has not been considered in existing investigations of sexual networking during the course of ART scale-up. Instead, most mathematical models of TasP have considered that sexual networks were formed either at random, or by mixing between different pre-defined risk groups (e.g., [5, 32–34]). Empirical investigations of sexual networks have mainly focused on preferences for partners of the same age, gender, educational level or ethnic group [35–43]. In the subpopulation of men who have sex with men, another form of homophily called “serosorting” has also been extensively investigated [44–53]. This is a behavioural strategy in which individuals preferentially select partners of the same HIV serostatus as them, so as to limit HIV transmission risks while possibly enabling unprotected sex.

### The emergence of ART clusters in sexual networks

**Fig 2:**
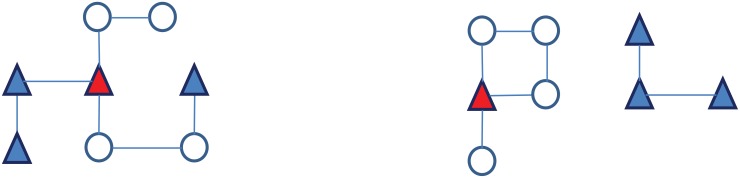
Illustration of the effects of ART homophily on HIV exposure in a population. HIV-negative individuals appear in empty circles, while people living with HIV (PLWH) are represented by triangles. ART patients appear in blue triangles, whereas other (untreated) PLWH appear in red triangles. In panel A) the network is formed at random, in panel B) the network is formed based on ART homophily: all HIV-negative individuals are connected only to the one PLWH not on ART, and the 3 ART patients are connected together in an ART cluster.

Despite this lack of attention, ART homophily has the potential to profoundly modify the structure of sexual networks, and as a result the level of exposure to HIV among HIV-negative individuals in the population. In the absence of ART homophily (Fig 2A), ART patients are likely disseminated throughout the sexual network and may come into contact with both untreated PLWH and HIV-negative individuals. In the presence of ART homophily however, ART patients may form “ART clusters”, which are subsets of the sexual network in which the density of ART patients is much higher than in the rest of the network. In the extreme case where ART patients only form relationships with other ART patients, these clusters may become disconnected from the rest of the network (Fig 2B). As ART clusters emerge, the extent of sexual contact between ART patients and other population members declines. At the same time, the number of relations between untreated (and thus likely more infectious) PLWH and HIV-negative individuals may increase to offset the reduced availability of ART patients to form new relationships with members of the other population groups (Fig 2B). If the level of exposure to HIV changes in a population during the course of ART scale-up as a result of ART homophily, then the impact of ART on HIV incidence may be modified.

ART clusters should not be confused with HIV transmission clusters. The latter typically refer to PLWH who share similar HIV genome sequences, as a result of being closely related in the HIV transmission network. ART clusters, on the other hand, may include relationships between PLWH who are very far removed from one another in the transmission network. Hence, ART clusters may connect PLWH whose HIV genome sequences are highly dissimilar.

We constructed an exploratory mathematical model of the impact of ART on HIV incidence that includes the possibility of ART homophily. This is not a formal network model, nor does it aim to describe the long-term dynamics of an epidemic. Instead, we solely used this model to assess whether the emergence of ART homophily in sexual networks may affect the effectiveness of ART in preventing HIV infections.

## Methods

### Model structure

We developed a static mathematical model for the population-level impact of ART on HIV incidence. The model population contains four interacting subgroups: HIV-negative individuals, PLWH who are unaware of their infection (and thus are not treated and remain infective), PLWH who are aware of their infection but are not treated (and thus remain infective), and PLWH who are on ART (“ART patients”). The latter group becomes less infective but the suppressive effect of ART on infectivity depends on their level of adherence to ART (see below).

Model parameters include the population prevalence of HIV (noted *h*), the fraction of PLWH who know that they are HIV-positive (*d*), the uptake of ART among diagnosed PLWH (*a*) and the reduction in HIV transmission associated with ART in serodiscordant couples (*r*). The coverage of ART, i.e. the proportion of PLWH who are on ART is defined as *da*. We define *r* as the HIV incidence in serodiscordant couples in which the infected partner has initiated ART divided by the HIV incidence among serodiscordant couples in which the infected partner has not initiated ART. It is thus an incidence rate ratio: the lower the value of *r*, the larger the reduction in HIV incidence associated with ART in serodiscordant couples. The value of *r* depends primarily on the extent to which the infected partner in the couple adheres to ART. The model also accounts for the average number of partners per time unit (*p*), the average number of unprotected sex acts per partnership (*s*), and the average HIV transmission probability per unprotected sex act (*i*).

The key parameters describing sexual networking patterns in this interacting population are *n*, the degree of serosorting and *m*, the degree of ART homophily. Both parameters vary between 0 and 1. When *n* = *m* = 0, relationships in the population are formed at random with respect to HIV status and ART status. This means that relationships between different population subgroups are formed proportionately to the prevalence of each of these subgroups in the population. When *n* = 1 and *m* = 0, diagnosed PLWH exclusively form partnerships with other diagnosed PLWH, but among those, PLWH on ART do not preferentially seek partners who are also on ART. Instead, partnerships between PLWH who are on and off ART are formed proportionately to the relative size of these groups. When *m* = 1, ART patients exclusively form partnerships with other ART patients, resulting in the emergence of ART clusters that are separated from the rest of the sexual network, as in Fig 2B. When 0 < *n* < 1, diagnosed PLWH have preferences for being in relationships with other diagnosed PLWH, but still have a proportion of their relationships with other PLWH who are not yet aware of their status and with HIV-negative people. Likewise, when 0 < *m* < 1, ART patients have preferences for being in relationships with other ART patients, but still have a proportion of their relationships with others who are not ART patients.

### Assumptions

To simplify the analysis, we assumed that there are no differences in infectivity between PLWH who are aware or unaware of their HIV-positive status if they are not treated. Similarly, the infectivity of ART patients depends solely on their level of adherence to ART. We further assumed no risk compensation: ART initiation does not lead to changes in the number of sexual partners individuals have, nor does it affect condom use or other determinants of the HIV transmission process within couples. Finally, we assumed homogeneity in sexual activity: every member of the population has the same number of partners and each sexual partnership entails the same number of sex acts.

### Model equations

In serodiscordant couples, when the HIV-positive partner is not on ART, the per-partnership probability of HIV transmission, noted *t*, is 1 − (1 − *i*)^*s*^. When the HIV-positive partner is on ART, this probability, noted *t*_*ART*_, is reduced because of the factor *r*. It is then equal to 1 − (1 − *i*)^*rs*^. The rates at which HIV-negative people form relationships with diagnosed PLWH on and off ART are *phda*(*m* − 1)(*n* − 1) and *phd*(1 − *a*)(1 − *n*) respectively. The rates of relationship formation with other HIV-negatives and undiagnosed PLWH are proportional to the size of the populations of HIV-negatives and undiagnosed PLWH: 1 − *h* and *h*(1 − *d)*, respectively. Taken together, these four rates sum to *p*, the annual number of partners that individuals have in our model. The HIV incidence rate, *I*, can then be calculated from these partner turnover rates and their associated per-partnership probabilities of HIV transmission (S1 File). Lastly, the population-level impact of ART on HIV incidence, *I*/*I*_*noART*_ − 1, is defined as the relative change in the HIV incidence rate, associated with ART. In this formulation, *I*_*noART*_ is calculated as *I* except that none of the PLWH are on ART (*a* = 0).

*Values of model parameters*: We considered three HIV prevalence levels (1%, 10% and 35%). The case where *h* = 1% represents situations typical of concentrated epidemics, whereas *h* = 35% represents situations common in certain demographic strata of hyperendemic settings [54–57]. Percentages of PLWH that are aware of their HIV status (*d*) and ART uptake among these diagnosed PLWH (*a*) ranged from 50% to 90% [58, 59], under the assumption of immediate, unconditional access to ART, as currently being piloted in TasP trials [60–63]. The upper bounds for the ranges of *a* and *d* in our model are also consistent with the new 90-90-90 UNAIDS targets for ART scale-up. The range of values for (*r*), i.e. the reduction in HIV incidence in serodiscordant couples associated with ART, was derived from a recent systematic review of prospective studies of the effect of ART on HIV transmission [9]. In contexts of high adherence to ART, the incidence of HIV in serodiscordant couples in which the PLWH is on ART, is reduced by 96% (i.e. *r* = 0.04) compared to similar couples in which the PLWH has not initiated ART. In contexts of low adherence to ART, the incidence of HIV in such serodiscordant couples is only reduced by 66% (i.e. *r* = 0.34). There are currently no quantitative data on the extent of serosorting in generalized epidemics in heterosexual networks, or on the level of ART homophily in sexual networks. We thus analyzed situations in which the parameters *n* and *m* vary between 0 (no homophily) and 1 (perfect homophily).

### Model analysis

Our goal in this exploratory model is to assess whether the addition of a parameter describing a previously understudied sexual networking pattern (i.e., ART homophily) might modify the impact of ART on HIV incidence. We thus do not seek to characterise the dynamics and steady state properties of the HIV transmission system described above.

We began by conducting a one-way analysis to assess how each model parameter relates to the population-level impact of ART under the assumption of random mixing with respect to ART status (*m* = 0). We then explored how the presence of ART homophily modifies the impact of ART on HIV incidence. To do so, we computed the impact of ART on HIV incidence (*I*/*I*_*noART*_ − 1) under 2 scenarios: a baseline scenario without any ART-homophily (m = 0) and a test scenario with perfect ART homophily (m = 1). The ratio of these two estimates of ART impact is the modification factor associated with ART homophily. When it is above 1, ART homophily enhances the impact of ART on HIV incidence, compared to a situation in which ART patients select partners at random. When this modification factor is below 1, then ART homophily reduces the impact of ART on HIV incidence. We conducted another one-way analysis to assess how each model parameter listed in Table 1 affects this modification factor.

**Table 1:** Range of parameters values explored in mathematical model.

Parameter	Description	Range in one-way analysis	Values used in multi-way analysis
***h***	HIV prevalence	1%– 35% (10%)	1%, 10% and 35%
***d***	Proportion of PLWH who are diagnosed	25%– 90%	25%– 90%
***a***	ART uptake among diagnosed PLWH	25%– 90%	50%
***p***	Annual number of partners	0.05–100	0.5
***s***	Number of unprotected sex acts per relationship	1–500	200
***i***	Per sex act HIV transmission probability	0.001–0.01	0.005
***r***	Incidence rate ratio associated with ART in serodiscordant couples^1^	0.04–0.34	0.04–0.34
***m***	ART assortativity index^2^	0–1	0; 1
***n***	HIV serosorting index^3^	0–1	0; 0.5

Notes:

^**1**^ Lower values of *r* are associated with higher adherence to ART among PLWH in serodiscordant couples. Lower values of *r* are thus also associated with larger reductions in HIV incidence in serodiscordant couples.

^2^ the ART assortativity measures the proportions of relationships of ART patients that are with other ART patients.

^3^ The HIV serosorting index measures the proportion of relationships of PLWH (regardless of diagnosis or treatment status) that are with other PLWH.

Finally, we conducted a multi-way analysis to identify the epidemiological and programmatic conditions under which ART homophily may enhance or reduce the impact of ART on HIV incidence. Specifically, we calculated the modification factor associated with ART homophily for each combination (*h; d; a; r; n*) of HIV prevalence (*h*), HIV diagnosis (*d*), ART uptake (*a*) intra-couple effectiveness of ART (*r*), and serosorting (*n*) defined in Table 1. Since the parameters *d* and *r* are allowed to vary over a range of values, the results from the multi-way analysis are presented as contour plots.

To facilitate further exploration of the behaviour of the model and strengthen intuition for the model results, we developed an online app (https://artclustering.shinyapps.io/ModelExploration) that interactively illustrates how the sexual network that connects the four population subgroups, and the impact of ART on HIV incidence, change as a function of ART-homophily and the other model parameters.

## Results

### ART impact in the absence of ART homophily

**Fig 3:**
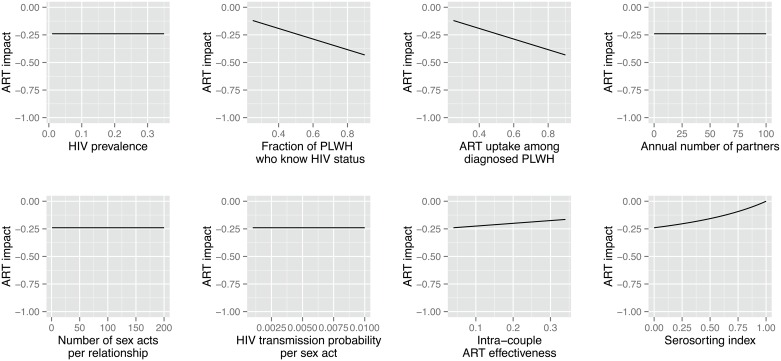
Population-level impact of ART on HIV incidence, as a function of the model parameters (one-way analysis). ***Notes*:** The impact of ART on HIV incidence is defined as the relative change in the HIV incidence rate, associated with ART (*I*/*I*_*noART*_ − 1). An ART impact of 0 thus indicates no effects of ART on HIV incidence, whereas an ART impact of -1 indicates that ART eliminates HIV incidence.

In sexual networks without ART-homophily (*m* = 0), in our model, the impact of ART on HIV incidence only depends on the proportion of PLWH aware of their status (*d*), ART uptake (*a*) the intra-couple ART effectiveness (*r*), and the extent of serosorting among PLWH (*n*). The annual number of partners (*p*), the average number of unprotected sex acts per relationship (*s*) and the per sex act probability of HIV transmission (*i*) do not affect the impact of ART on HIV incidence in this model (Fig 3). This does not mean that these parameters do not influence HIV incidence. Indeed, HIV incidence increases with increasing values of *p*, *s* and *i*, but the relative change in HIV incidence when comparing scenarios with and without ART is unaffected. Henceforth, these parameters were not included in the multi-way analysis (Table 1). Serosorting among PLWH reduces the impact of ART on HIV incidence. In particular, when *n* = 1, all the partnerships formed between HIV-negative people and PLWH are necessarily with undiagnosed PLWH. In this context, the impact of ART drops to zero.

### Modification factor associated with ART homophily

**Fig 4:**
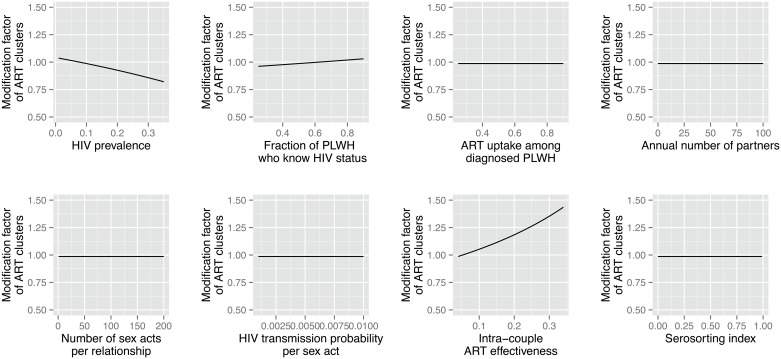
Effects of model parameters on the modification factor of the impact of ART associated with ART homophily. *Notes*: The modification factor is calculated by dividing the estimated impact of ART in a context with perfect ART homophily (*m* = 1) by the estimated impact of ART in a context without any ART homophily (*m* = 0).

Fig 4 indicates that the modification factor associated with *m*, i.e. the relative change in the impact of ART that results from the presence of ART homophily, depends on HIV prevalence (*h*), the fraction of PLWH aware of their status (*d*), and adherence to ART (*r*), but not on other model parameters. In particular, the modification factor appears largest in settings where adherence to ART is low. Changes in the prevalence of serosorting among PLWH, on the other hand, have no effects on the modification factor associated with ART homophily.

Analysis of the model’s equations (S1 File) suggests that ART clusters will weaken the prevention benefits of ART if the subpopulation of undiagnosed PLWH is larger than *r* times the combined size of the subpopulations of HIV-negative people and undiagnosed PLWH. Conversely, ART clusters will augment the impact of ART on HIV incidence if the opposite is the case. The strongest synergistic effect of ART homophily on the impact of ART on HIV incidence is thus achieved when *r* is very large, *h* is small and *d* is large, i.e. low ART adherence in a population with low HIV prevalence but a high fraction of PLWH being diagnosed. On the other hand, ART homophily can reduce the prevention benefits of ART if it occurs in a context of high HIV prevalence where not many PLWH are diagnosed (small *d*), but treatment effectiveness is excellent (small *r*).

**Fig 5:**
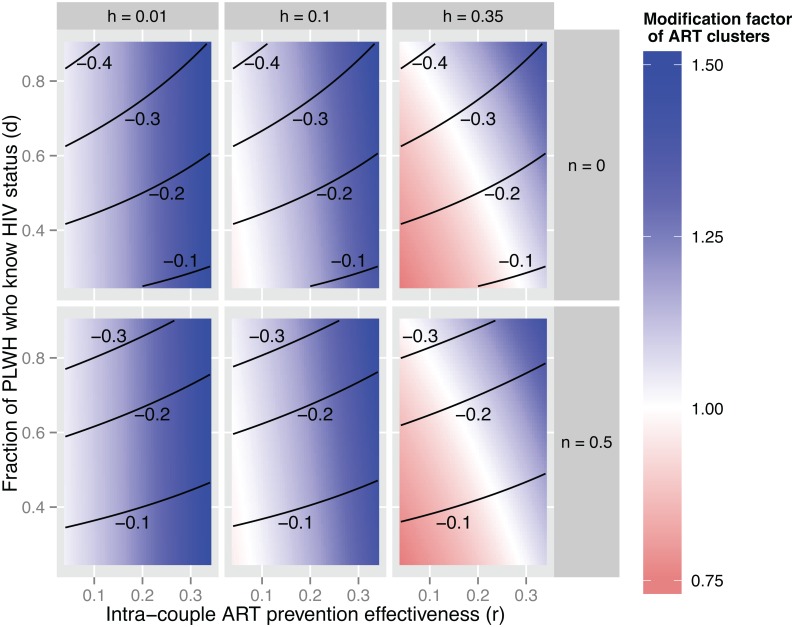
The effect of ART homophily and serosorting on the population-level impact of ART on HIV incidence, by levels of HIV prevalence (*h*), fraction of PLWH who are aware of their HIV status (*d*) and intra-couple effectiveness of ART (*r*). Contour lines indicate the impact of ART on HIV incidence in the absence of ART clusters (*m* = 0). Color-coding indicates the modification factor of ART clusters: the factor by which the ART impact on HIV incidence increases (> 1 in blue) or decreases (< 1 in red) when comparing the case of *m* = 1 to the case of *m* = 0. For example, in the darkest blue areas, the impact of ART on HIV incidence is 50% greater in the presence of perfect ART homophily (*m* = 1) than it would have been if networks were formed without any ART homophily (*m* = 0). The uptake of ART among diagnosed PLWH (*a*) was fixed at 50% in all model scenarios shown.

In populations with HIV prevalence ≈ 1% (Fig 5), ART homophily thus increases the impact of ART on HIV incidence relative to a baseline without ART-homophily for virtually all combinations of model parameters. ART homophily particularly improves the impact of ART on HIV incidence, however, if ART patients do not strictly adhere to treatment. For example, in settings with low ART adherence among ART patients (*r* = 0.34), ART homophily increases the impact of ART on HIV incidence by close to 50%, regardless of other model parameters. On the other hand, if adherence is high (*r* = 0.04), then ART homophily only increases the impact of ART by 10% relative to a situation in which networks are formed at random.

Similar results are obtained in populations with HIV prevalence ≈ 10%, but the potential effects of ART homophily on the population-level impact of ART vary more widely when HIV prevalence ≈ 35% (Table 2). In such hyper-endemic populations, ART homophily may also improve the impact of ART on HIV incidence when adherence to ART is low. For example, in contexts with high proportions of PLWH aware of their infection, but low adherence to ART (upper right corner), then ART homophily increases the impact of ART by close to 50% relative to similar contexts in which networks would be formed at random with respect to ART status. On the other hand, if ART adherence is high, then the emergence of ART clusters can reduce the expected impact of ART on HIV incidence. For example, in populations where a limited proportion of PLWH are diagnosed, ART homophily may reduce the impact of ART by close to 25% (lower left corner).

**Table 2:** Hypotheses and projections for the main model scenarios (see also Fig 5).

Hypotheses	Concentrated HIV epidemics	Generalised HIV epidemics	Hyperendemic HIV epidemics
HIV prevalence (h)	≈ 1%	≈ 10%	≈ 35%
Intra-couple ART prevention effectiveness (r)	Available evidence suggests that ART confers a similar prevention benefit to MSM as to heterosexual serodiscordant couples	A systematic review and meta-analysis of prospective studies among serodiscordant couples suggests that ART may reduce HIV incidence by 66% to 96%	A systematic review and meta-analysis of prospective studies among serodiscordant couples suggests that ART may reduce HIV incidence by 66% to 96%
Fraction of PLWH who know HIV status (d)	In some concentrated HIV epidemics (e.g. MSM in Switzerland), more than 75% of PLWH know their status, but in other key populations (e.g. people who inject drugs) in Asia, this fraction may be far below 50%	About half of all PLWH in Sub-Saharan Africa know their status, but knowledge of HIV status is lower among youth and men.	About half of all PLWH in Sub-Saharan Africa know their status, but knowledge of HIV status is lower among youth and men.
Fraction of ART patients among *diagnosed* PLWH (a)	Estimates range from 40% in the USA to 77% in Australia	Close to 90% of PLWH who know their status in sub-Saharan Africa are receiving ART.	Close to 90% of PLWH who know their status in sub-Saharan Africa are receiving ART.
Level of HIV serosorting (n)	Estimates for the prevalence of serosorting among MSM range between 10% and 40%	There are currently no quantitative data on the extent of serosorting in generalized epidemics	There are currently no quantitative data on the extent of serosorting in hyperendemic epidemics
**Projections**			
Effect of ART clusters	ART clusters may enhance impact of ART on HIV incidence by up to 50% if intra-couple ART prevention effectiveness is poor	ART clusters may enhance impact of ART on HIV incidence by up to 50% if intra-couple ART prevention effectiveness is poor	ART clusters may reduce ART impact by about 25% if intra-couple ART prevention effectiveness is high
Effect of HIV serosorting	HIV serosorting reduces the impact of ART on HIV incidence, but does not influence the relative effect of ART clusters	HIV serosorting reduces the impact of ART on HIV incidence, but does not influence the relative effect of ART clusters	HIV serosorting reduces the impact of ART on HIV incidence, but does not influence the relative effect of ART clusters

## Discussion

Unlike other sexual mixing patterns such as serosorting [44, 45, 64, 65] and mixing between and across subgroups with varying levels of sexual risk behaviour [4, 66], ART homophily has not previously been considered in mathematical models of the effectiveness of ART for HIV prevention. This is a significant gap since the ART status of potential partners can play a significant role in relationship decisions among ART patients [27].

Using an exploratory mathematical model, we showed that ART homophily may modify the prevention impact of ART in a complex manner, depending simultaneously on the performance of HIV testing and treatment programmes (e.g., coverage of HIV testing and ART adherence) and the epidemiological context (HIV prevalence). Our analysis further showed how the effect of ART homophily is different from that of serosorting.

In concentrated epidemics and in generalized epidemics where HIV prevalence is no more than 10%, ART homophily enhances the impact of ART on HIV incidence. On the other hand, the impact of ART clusters may be more complex in hyperendemic settings where the HIV prevalence among certain gender-age strata may reach or even exceed 35% [54–57]. In such settings, ART homophily also enhances the impact of ART on HIV incidence when HIV status awareness among PLWH (*d*), and therefore ART coverage (*da*) are high, and adherence to ART is low. This is so because ART patients who do not adhere to treatment remain infective. In that case, ART clusters provide indirect protection to HIV-negative individuals by limiting their contact with potential sources of HIV transmission.

In contrast, in hyperendemic settings where HIV status awareness (and hence ART coverage) is low but adherence to ART is high, ART homophily may reduce the impact of ART on population-level HIV incidence. In this scenario, highly-adherent ART patients (who are significantly less infective than other PLWH) would have helped interrupt chains of HIV transmission in sexual networks connected to HIV-negative individuals. Instead, because of ART clusters, HIV-negative individuals are more likely to come into contact with undiagnosed (and untreated) and thus more infective PLWH. Since the combination of high ART adherence and low ART coverage characterizes most current ART programmes in sub-Saharan countries, ART may at the moment have a lower impact on HIV incidence than estimated by standard mathematical models without ART homophily [67, 68].

Our model projections provide *qualitative* insight into why ART clusters may affect the impact of ART on HIV incidence. Our goal was not, however, to make precise, quantitative statements about the expected effects of ART clustering for specific populations or geographical regions. Accordingly, our analysis has several important limitations. Firstly, empirical investigations of ART homophily (the value of *m*) are required to obtain a quantitative understanding of the effect of ART homophily on the effectiveness of ART. Such data are not currently available, so we let our model span the entire range of possible parameters. Questions about the ART status of one’s sexual partners should thus be included in studies of sexual behaviours conducted among ART patients, as is already the case in the MaxART study, an ongoing implementation study of early access to ART for all PLWH in Swaziland [69]. Secondly, our model only considered situations in which there was no risk compensation among ART patients. Future investigations of ART homophily should incorporate interactions between risk compensation and the emergence of ART clusters. Thirdly, we only considered ART-related sexual mixing patterns. But partner choices could be more complex in the context of combination prevention, in which ART is scaled-up alongside other HIV prevention interventions such as medical male circumcision. Fourthly, our model did not include the acute phase of HIV infection, during which PLWH have an elevated viral load and are highly infectious [70, 71]. Incorporating an acute phase with increased infectiousness and (near) zero coverage of ART during this phase would change the model’s estimate for the impact of ART on HIV incidence. However, given that ART coverage and ART clustering are likely extremely low among people in the acute phase of HIV infection in both the base scenario and comparison scenario, adding this phase to the model would not affect the *relative* impact of ART on HIV incidence. We also only considered populations in which all individuals have the same number of sexual partners, even though heterogeneity in sexual activity and other forms of sexual network structure influence the impact of ART on HIV incidence [4]. It is less clear, however, if these network attributes also affect the relative effect of ART clusters on the HIV prevention benefits of ART. Moreover, assortative mixing, by sexual activity level, age, or other demographic or behavioural attributes, may lead indirectly to apparent serosorting. While our model assumes homogeneous sexual activity, it does allows for an arbitrary degree of serosorting among HIV-positive individuals, without making specific assumptions about the underlying dynamics that led to serosorting. Therefore, our model is not necessarily at odds with a more complicated model in which heterogeneity in sexual activity and some degree of assortative mixing by sexual activity level leads to serosorting. Finally, we focused on exploring the instantaneous change in the effectiveness of ART in reducing HIV incidence following the emergence of ART homophily. In doing so, we did not fully characterise the dynamics of the HIV transmission system we analysed, nor did we investigate its equilibrium properties. Future research on the impact of ART homophily should thus use more refined modelling strategies including network models or compartmental models, which permit addressing these questions.

## Conclusions

We show that ART homophily in sexual networks can significantly modify the population-level impact of ART on HIV incidence, even in the absence of risk compensation. But the magnitude and the direction of this modification depends on many other variables: HIV prevalence, coverage of HIV testing and ART, ART adherence and the level of serosorting. Our results suggest that the mathematical models that are being used to estimate the current and future impact of TasP programmes should be amended to take into account the possible emergence of ART clusters in sexual networks during the course of ART scale-up. This inclusion would yield a better estimation of expected reductions in HIV incidence due to accelerated access to ART, as well as a better understanding of observed time trends in HIV incidence after ART scale-up. In contexts where ART clusters could enhance the impact of ART on HIV incidence (e.g. low prevalence settings), measures to promote the formation of ART clusters (e.g. by organising ART initiation and post-initiation follow-up for couples) could constitute useful complementary interventions in combination HIV prevention programmes.

## Supporting Information

S1 File Derivation of model equations and outcomes. The probability of HIV transmission per sex act when the HIV-positive partner is on ART (*t*_*ART*_) can be derived from the incidence rate ratio (*r*) in serodiscordant couples on vs off ART. From the model equations, the population-level impact of ART on HIV incidence can be calculated, in the case of random mixing, as well as in the case of serosorting and ART clustering.
(DOCX)
