# Supplementary material for: Beyond Risk Compensation: Clusters of Antiretroviral Treatment (ART) Users in Sexual Networks Can Modify the Impact of ART on HIV Incidence
Source: PLoS One. 2016 Sep 22;11(9):e0163159. doi: 10.1371/journal.pone.0163159 (PMC5033240; doi:10.1371/journal.pone.0163159)
Supplement: S1 File — The probability of HIV transmission per sex act when the HIV-positive partner is on ART (tART) can be derived from the incidence rate ratio (r) in serodiscordant couples on vs off ART. From the model equations, the population-level impact of ART on HIV incidence can be calculated, in the case of random mixing, as well as in the case of serosorting and ART clustering. (DOCX) [file pone.0163159.s001.docx]

**Supplementary Material**

**A. Deriving the probability of HIV transmission per sex act when the HIV-positive partner is on ART (**$\boldsymbol{t}_{\boldsymbol{ART}}$**) from the incidence rate ratio (**$\boldsymbol{r}$**) in serodiscordant couples on vs off ART.**

The risk of HIV transmission per sex act in the absence of ART, $i$, can be viewed as the result of a continuous force of infection $f$, maintained for a short period of time $\delta_{i}$ (the average interval between two consecutive sex acts):

$$i=1-e^{-f\delta_{i}}$$

Likewise, the risk of HIV transmission per sex act while the HIV positive partner is on ART, $i_{ART}$, is the result of a reduced force of infection. The incidence rate ratio, $r$, expresses the relative reduction in this force of infection:

$$i_{ART}=1-e^{-fr\delta_{i}}$$

Hence:

$$ln\left( 1-i \right)=-f\delta_{i}$$

$$ln\left( 1-i_{ART} \right)=-fr\delta_{i}$$

$$ln\left( 1-i_{ART} \right)=ln\left( 1-i \right)r$$

$$1-i_{ART}=\left( 1-i \right)^{r}$$

$$i_{ART}=1-\left( 1-i \right)^{r}$$

Probability of transmission per relationship ($s$ unprotected sex acts):

$$t_{ART}=1-\left( 1-i_{ART} \right)^{s}=1-\left( \left( 1-i \right)^{r} \right)^{s}$$

$$t_{ART}=1-\left( 1-i \right)^{rs}$$

**B. Calculation of the population-level impact of ART on HIV incidence in the case of random mixing (**$\boldsymbol{m=0}$**,** $\boldsymbol{n=0}$**)**

If sexual mixing is random with respect to HIV status and ART status (i.e., $m=0$ and $n=0$), then the fraction of relationships formed by HIV-negative people with PLWH on and off ART is equal to $hda$ and $h\left( 1-da \right)$, respectively.

In the absence of any ART programme, the HIV incidence rate at the population level, $I$, is simply $-ln\left( 1-t \right)ph$. If some PLWH are on ART ($da>0$), the HIV incidence rate becomes:

$$I=-ln\left( 1-t_{ART} \right)phda-ln\left( 1-t \right)ph\left( 1-da \right)$$

The population-level impact of ART on HIV incidence, $I/{I_{noART}}-1$, is defined as the relative change in the HIV incidence rate, associated with ART, whereby $I_{noART}$ follows the same calculation as $I$ except that this time no one is on ART ($a=0$).

**C. Calculation of population-level impact of ART on HIV incidence in the case of serosorting (**$\boldsymbol{n>0}$**) and ART clustering (**$\boldsymbol{m>0}$**).**

If some degree $n$ of serosorting is introduced, PLWH who know their HIV status will form more relationships with other diagnosed PLWH. If some degree $m$ of ART clustering is also introduced, the subset of PLWH on ART will still have the same fraction of their relationships with other diagnosed PLWH as diagnosed PLWH not on ART, but in addition, they will disproportionally favour PLWH who are also on ART, when forming new relationships.

These relative shifts in relationship preferences for diagnosed PLWH and PLWH on ART subgroups, lead to compensatory shifts for HIV-negative people. The fraction of their total number of relationships that are formed with PLWH on ART is no longer $hda$ but $hda\left( 1-n \right)\left( 1-m \right)$, and the fraction with PLWH who are diagnosed but not on ART is $hd\left( 1-a \right)\left( 1-n \right)$.

To compensate for the reduced availability of diagnosed PLWH off ART as a result of serosorting and the reduced availability of diagnosed PLWH on ART as a result of serosorting and ART clustering, HIV-negative people will form more relationships with other HIV-negative people and undiagnosed PLWH, proportional to their respective subpopulation group size.

The total fraction of their relationships with diagnosed PLWH is:

$$hda\left( 1-n \right)\left( 1-m \right)+hd\left( 1-a \right)\left( 1-n \right)=hd\left( 1-n \right)-hda\left( 1-n \right)m$$

Of all people in the population, the respective fractions $h\left( 1-d \right)$ and $1-h$ are undiagnosed PLWH and HIV-negative people. Hence, the fraction of HIV-negative people’s relationships formed with undiagnosed PLWH is:

$$\frac{h\left( 1-d \right)}{\left( h\left( 1-d \right)+\left( 1-h \right) \right)\left( 1-\left( hd\left( 1-n \right)-hda\left( 1-n \right)m \right) \right)}$$

The fraction of HIV-negative people’s relationships formed with other HIV-negative people is:

$$\frac{1-h}{\left( h\left( 1-d \right)+\left( 1-h \right) \right)\left( 1-\left( hd\left( 1-n \right)-hda\left( 1-n \right)m \right) \right)}$$

The HIV incidence rate can now be calculated as:

$$I=-ln\left( 1-t_{ART} \right)phda\left( 1-n \right)\left( 1-m \right)$$

$$-ln\left( 1-t \right)phd\left( 1-a \right)\left( 1-n \right)$$

$$-ln\left( 1-t \right)p\left( \frac{h\left( 1-d \right)}{h\left( 1-d \right)+\left( 1-h \right)}\left( 1-\left( hd\left( 1-n \right)-hda\left( 1-n \right)m \right) \right) \right)$$

As was the case for random mixing with respect to HIV status and ART status, the population-level impact of ART on HIV incidence, $I/{I_{noART}}-1$, is defined as the relative change in the HIV incidence rate, associated with ART, whereby $I_{noART}$ follows the same calculation as $I$ except that this time no one is on ART ($a=0$).

$$I_{noART}=-ln\left( 1-t \right)phd\left( 1-n \right)$$

$$-ln\left( 1-t \right)p\left( \frac{h\left( 1-d \right)}{h\left( 1-d \right)+\left( 1-h \right)}\left( 1-\left( hd\left( 1-n \right) \right) \right) \right)$$

From the equations above, we see that both the incidence, *I*, and the population-level impact of ART on HIV incidence, $I/{I_{noART}}-1$, change linearly with $m$, but the direction in which they change depends on $h$, $d$ and $r$ (because $r$ determines how different $t_{ART}$ is from $t$.

The parameter $m$ subtracts $m$ times $-ln\left( 1-t_{ART} \right)phda\left( 1-n \right)$ from the incidence, but also adds $m$ times $-ln\left( 1-t \right)\frac{h\left( 1-d \right)}{h\left( 1-d \right)+\left( 1-h \right)}phda\left( 1-n \right)$. After removing the scaling factor$phda\left( 1-n \right)$, we see that only $t$, $t_{ART}$, $h$ and $d$ remain.

Since $-ln\left( 1-t_{ART} \right)$ is $r$ times smaller than $-ln\left( 1-t \right)$, the question becomes:

Is $\frac{h\left( 1-d \right)}{h\left( 1-d \right)+\left( 1-h \right)}$ smaller or larger than $r$? We can also find the turning point, by expressing one parameter as a function of the other two: when $\frac{h\left( 1-d \right)}{h\left( 1-d \right)+\left( 1-h \right)}=r$ then $d=\frac{h-r}{h-hr}$ . For instance, when the HIV prevalence is 35% and the incidence rate ratio associated with ART among serodiscordant couples is 0.04, it is sufficient that less than 92% of PLWH are aware of their status for ART clustering to have an impeding effect on the prevention benefits of ART. For the same ART incidence rate ratio but a background prevalence of 10%, status awareness among PLWH needs to drop below 62% before ART begins to weaken the impact of ART on HIV incidence.

In other words, ART clustering will weaken the prevention benefits of ART if the subpopulation of undiagnosed PLWH is more than *r* times larger than the sum of the subpopulations of HIV-negative people and undiagnosed PLWH. Conversely, ART clustering will augment the impact of ART on HIV incidence if the opposite is the case. From this, it follows that the strongest synergistic effect of ART clustering on the impact of ART on HIV incidence is achieved when $r$ is very large, $h$ is small and $d$ is large, while ART clustering can reduce the prevention benefits of ART if it occurs in a context of high HIV prevalence where not many PLWH are diagnosed (small $d$), but treatment effectiveness is excellent (small $r$).

Serosorting results in a smaller fraction of relationships being formed between HIV-negative people and PLWH, regardless of all the other parameters. Because PLWH on ART are by definition aware of their HIV status, and therefore subject to a level $n$ of serosorting, serosorting results in a reduced incidence rate ratio between the ART and no ART scenario. In the limit of $n=1$, all contacts that HIV-negative people have with PLWH are necessarily in the absence of ART, such that the impact of ART drops to zero. Note that an analogy with the case of perfect ART clustering ($m=1$) cannot be drawn, by definition, because for ART clustering to be possible, there must be at least some level of ART uptake ($da>0$).
